# Niches and Interspecific Competitive Relationships of the Parasitoids, *Microplitis prodeniae* and *Campoletis chlorldeae*, of the Oriental Leafworm Moth, *Spodoptera litura*, in Tobacco

**DOI:** 10.1673/031.010.1001

**Published:** 2010-03-02

**Authors:** Zhong-Shi Zhou, Ze-Peng Chen, Zai-Fu Xu

**Affiliations:** ^1^ Department of Entomology, College of Resources and Environment, South China Agricultural University, Guangzhou, 510642, The People's Republic of China; ^2^ State Key Laboratory for Biology of Plant Diseases and Insect Pests, Institute of Plant Protection, Chinese Academy of Agricultural Sciences, Center for Management of Invasive Alien Species, Ministry of Agricultural, P. R. China, Beijing 100081, China; ^3^ Guangdong Company of Tobacco, Guangzhou, 510030, The People's Republic of China

**Keywords:** *Nicotiana tabacum*, *Helicoverpa assulta*, *Helicoverpa armigera*, biological control, niche

## Abstract

Both *Microplitis prodeniae* Rao and Chandry (Hymenoptera: Bracondidae) and *Campoletis chlorideae* Uchida (Hymenoptera: Ichnumonidae) are major parasitoids of *Spodoptera litura* (Fabricious) (Lepidoptera: Noctuidae) in tobacco, *Nicotiana tabacum* L. (Solanales: Solanaceae) at Nanxiong, Guangdong Province, South China. The niches and interspecific competition relationships of the two species were studied. The results show that the competition between the two species for spatial and food resources was very intense, and *C. chlorideae* was always dominant when the two species compete for spatial and food resources in different periods. Thus *C. chlorideae* may drive *M. prodeniae* away when they occupy the same spatial or food resource. The adaptability of *C. chlorideae* to the environment in the tobacco fields may be greater than that of *M. prodeniae*, so *C. chlorideae* can maintain a higher population compared to that of *M. prodeniae*.

## Introduction

In nature, some species live together in the same or in similar niches because they have one or several kinds of similar behaviors, thus one species often conflicts with the other one for food resources. Interspecific competition is defined as a reduction in individual fecundity, survival, or growth as a result of exploitation of resources or by interference with individuals of another species (Begon et al. 1996). A superior species may exclude an inferior one from parts of its fundamental niche or, in extreme cases, drive it to extinction for occupying spatial vertical resources or food resources (Wissinger 1992). Thus, some species often are in an inferior position when competition occurs among various species. The phenomenon is more obvious when food resources are in short supply.

According to the conventional niche theory, the primary determinant of interspecific competition is the overlap and the similarity of the resources used (niche overlap or niche similarity). Two species with highly similar fundamental niches (i.e. the niches potentially occupied in the absence of competitors) will often compete strongly with each other when they first meet (Duyck et al. 2004). This was also the conclusion reached by Shorrocks (1991), who found that two species of *Drosophila* coexisted in a spatially divided system, where the inferior species was eliminated in an undivided system.

Recent reviews have shown that interspecific competition is widespread among insects (Denno et al. 1995; Stewart 1996; Reitz and Trumble 2002). Competition is a universal phenomenon in nature, and in particular, among natural enemies. Natural enemies may conflict for occupying scope and prey, and a superior species of natural enemy may drive an inferior one away, thus limiting the population of the inferior species. Predators may kill their adversaries when they have conflict over spatial resources and food. In general, the adults of parasitoid wasps cannot kill their competitors, but they can drive competitors away for inhabiting advantageous niches and having access to more food (Bajpai et al. 2005). Some larvae of parasitoid wasps can, however, attack the larvae of their adversaries when two species parasitize the same host larvae (Vinson and Iwantsch 1980; Mackauer 1990; Tian et al. 2008).

In conventional agroecosystems, pest outbreaks are common because these simplified systems are species poor (Pimentel 1961), and agricultural pest suppression has been identified as an important ecosystem service that may be threatened by the loss of natural enemy biodiversity (Kruess and Tscharntke 1994; Wilby and Thomas 2002). These ideas suggest that managing for greater natural enemy biodiversity may improve pest suppression (Cardinale et al. 2003; Aquilino et al. 2005). Generally, biological control is rarely in conflict with biodiversity in agroecosystems, and thus biological control has been noticed by many researchers (Geoff and Steve 2000). *Microplitis prodeniae* Rao and Chandry (Hymenoptera: Bracondidae) and *Campoletis chlorideae* Uchida (Hymenoptera: Ichnumonidae) are both major parasitoid wasps in tobacco fields. In general, both *M. prodeniae* and *C. chlorideae* prefer to parasitize the young instar larvae of hosts, and the two parasitoids emerge from the body of the host when the host larvae is in the third instar. Thus they play an important role in the control of the oriental leafworm, *Spodoptera litura* (Fabricious) (Lepidoptera: Noctuidae) (Dang and Hung 1999; Liu et al. 2004; Bajpai et al. 2005), and regarded as the two species of candidate biological control agents for controlling the population of *S. litura* (Dang and Hung 1999; Bajpai et al. 2005). The biology and ecology of *C. chlorideae* has been extensively researched (Kumar et al. 2000; Murugan et al. 2000; Pandey et al. 2004; Yang et al. 2005; Yan and Wang 2006; Zhang et al. 2006), but a small number of studies have been probed into its biology and ecology since *M. prodeniae* was recorded as a parasitoid of *S. litura* larvae. In the search for alternatives to chemical insecticides, the utilization of these two species for protecting crop plants from *S. litura* has been assigned more importance in the recent decade (Dang and Hung 1999; Bajpai et al. 2005). However, most entomologists, ecologists and biologists have chosen only one of the two species as the target of study, and they have not probed into the ecological relationships between them, especially their niches and competition relationships. Therefore, we combined *M. prodeniae* with *C. chlorideae* and regard them as one entity. The objective of this study was to understand the ecological relationships of *M. prodeniae* and *C. chlorideae* in the field, and the results of this study provide a basis for the utilization of these two species as pest control for *S. litura*.

## Materials and Methods

### Study sites

This study was conducted in the tobacco field at the experimental farm of Nanxiong Research Institute of Tobacco, Nanxiong, Guangdong Province. There are 300 ha tobacco, *Nicotiana tabacum* L. (Solanales: Solanaceae), fields, and *S. litura* (Fabricious), *Helicoverpa assulta* Guenée and *Helicoverpa armigera* Hübner often occur together in tobacco fields in this research station. Several fields, where serious damage was caused by these three species, were taken as the target fields of investigation. The acreage of each of these experimental fields was about 6670 m^2^.

### Tobacco variety

*N. tabacum* 9601 variety was planted on 20 February 2006, and the density was 1.7–1.8 plants / m^2^.

### Spatial niches and temporal niches of *M. prodeniae* and *C. chlorideae*

Investigations were conducted from 10 May to 3 June in 2006 at the experimental farm of Nanxiong Research Institute of Tobacco. Spatial vertical resources in tobacco plants were divided into three even grades containing upper leaves, mid-leaves and underneath leaves, and temporal resources were divided into five even grades by investigation times (i.e. 10 May, 16 May, 22 May, 28 May and 3 June). Investigations took place once every five days. Second instar larvae of *S. litura* on the different positions of tobacco plants (*n* = 80) were collected by “random sample” every time, and the living larvae from the different positions were taken back to the laboratory. Then they were bred in separate cages, respectively, and fed fresh tobacco leaves every day. The parasitic rates of *M. prodeniae* and *C. chlorideae* on *S. litura* larvae from different positions were recorded when the parasitoid adults emerged from the bodies of *S. litura* larvae.

### Host resources of *M. prodeniae* and *C. chlorideae*

Investigations were conducted from 8 May to 19 June in 2006 at the experimental farm of Nanxiong Research Institute of Tobacco. Trophic resources were divided into three grades (i.e. *S. litura*, *H. assulta* and *H. armigera*). Investigation was carried out once every five days. Second instar larvae from *S. litura*, *H. assulta* and *H. armigera* (*n* = 80) were collected by “random sample” every time. The living larvae of these three species were taken back laboratory, and they were bred in different cages. Fresh tobacco leaves were given to them every day, and the parasitic rates of *M. prodeniae* and *C. chlorideae* on the larvae from these three species were recorded when the parasitoid adults emerged from the bodies of the larvae of these three species.

### Statistical Analyses

Regarding the ecological relationships of *M. prodeniae* and *C. chlorideae*, the niches and interspecific competition coefficients of the two species were compared. The ability of *M. prodeniae* and *C. chlorideae* to utilize resources was estimated by their niche breadths, by competition occurring between the two species, by niche overlap and niche similarity, and by the competitive degree of the two species to resources based on an interspecific competition coefficient. When the ability of a species to utilize resources is enhanced with the increase of the breadth value of its niche, competition may occur between it and another species when niche similarity proportion between the two species reveals a high value, and competitive degree between them strengthened with an increasing interspecific competition coefficient between the two species. The following formulae are recognized by ecologists, and they are applied for the evaluation of the interrelation between two species.

Niche breadth was calculated by *B* = 1 / (*s* 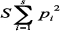). In this formula, *B* is niche breadth of species, *S* is the number of resource grades and *P_i_* is the fraction of all resources that belong to *i*-th grade resource used by the species (May 1975). Thus, with this formula, *P_i_* is the fraction of all spatial resources which belong to upper leaves or mid-leaves or underneath leaves occupied by *M. prodeniae* or *C. chlorideae* for the spatial niche calculation, *P_i_* is the fraction of all temporal resources which belong to *i*-th survey date used by *M. prodeniae* or *C. chlorideae* for the temporal niche calculation, and *P_i_* is the fraction of all host resources which belong to *S. litura*, *H. assulta*, or *H. armigera* parasitized by *M. prodeniae* or *C. chlorideae* for the trophic niche calculation.

Niche overlap and the proportion similarity of the niche were measured by 
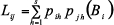
(*B_i_*) and 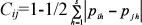, respectively, where *L_ij_* is the nicrie overlap index value and species *j* is overlapped by species *I*; *C_ij_* is the proportion similarity of the niches of species *i* and species *j*, *P_ih_* and *P_jh_* are the fractions of total resources which belong to *h*-th grade resource used by species *i* and by species *j*, respectively; and *B_i_* is the niche breadth of species *i* (Levins 1968; Cowll and Futuyma 1971). In this survey study, *P_ih_* and *P_jh_* are the fractions of total spatial resources which belong to *h*-th grade spatial resource occupied by *M. prodeniae* and by *C. chlorideae*, respectively, when the spatial niche overlap was calculated; *P_ih_* and *P_jh_* are the fractions of total temporal resources which belong to *h*-th survey date used by *M. prodeniae* and by *C. chlorideae*, respectively, when the temporal niche overlap was calculated; and *P_ih_* and *P_jh_* are the fractions of total host resources which belong to *h*-th grade host resources parasitized by *M. prodeniae* and *C. chlorideae* when the trophic niche overlap was calculated with these two formulas.

Temporal and spatial two-dimensional niche was measured by provided “multidimensional niche models” (May 1975). He suggested that the indices of the multidimensional niche be multiplied by the index of individual niche. Niche breadth value, niche overlap value and the proportion similarity of temporal and spatial two-dimensional niches were multiplied each by its individual counterpoint.

The interspecific competition coefficient was calculated by 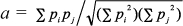 , where *a* is the interspecific competition coefficient and where *P_i_* and *P_j_* are the fractions of all resources which are used by species *i* and species *j*, respectively (May 1975). So *P_i_* and *P_j_* are the fraction of all spatial resources which are used by *M. prodeniae* and by *C. chlorideae*, respectively, when interspecific competition coefficient of spatial niche was calculated; and *P_i_* and *P_j_* are the fraction of all host resources which are parasitized by *M. prodeniae* and by *C. chlorideae*, respectively, when interspecific competition coefficient of trophic niche was calculated with this formula. The competition between species intensified if the interspecific competition coefficient increased.

A number of studies reveal that the intensity of competition between two species is not proportional to the niche overlap (Cowll and Futuyma 1971; Zhou et al. 2000; Qian et al. 2006), and the intensity of competition actually varied inversely with the niche overlap in a few cases (Qian et al. 2006). Two species with highly similar fundamental niches will often compete strongly with each other when they meet (Duyck et al. 2004). So it was assumed that competition between two different species would happen if the niche overlap index, the proportion similarity of niche and the interspecific competition coefficient are all high, or the niche overlap index is low, but the proportion similarity of niche and interspecific competition coefficient are still high.

## Results

### Temporal niches and spatial niches of *M. prodeniae* and *C. chlorideae*

**Figure 1:**
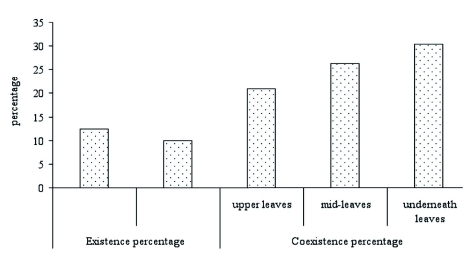
Existence model of *Spodoptera litura* and *Helicoverpa assulta* on tobacco. *Mp* and *Cc* are *Microplitis prodeniae* and *Campoletis chlorideae*, respectively. High quality figures are available online.

The spatial niche breadth value of *C. chlorideae* was slightly higher than that of *M. prodeniae*, and the spatial niche overlap value of *C. chlorideae* to *M. prodeniae* was close to that of *M. prodeniae* to *C. chlorideae*. The spatial niche proportion similarity between the two species was 0.7855. These results revealed that the active scope of *C. chlorideae* was more extensive than that of *M. prodeniae*, and the vertical distributions of the two species on spatial resources were similar. The temporal niche overlap value of *C. chlorideae* to *M. prodeniae* was significantly close to that of *M. prodeniae* to *C. chlorideae*. The temporal niche proportion similarity between them was 0.9850. The results suggested that *M. prodeniae and C. chlorideae* may occur together in tobacco fields at the same time. Temporal and spatial two-dimensional niche breadth of *C. chlorideae* revealed a relatively higher value compared with *M. prodeniae*, and the two species have a higher value in the two-dimensional niche proportion similarity. The interspecific competition coefficient between the two species was 0.8973, suggesting that severe competition happened between the two species because they each attempt to occupy more advantageous spatial vertical resources than the other (Table 1). In addition, the results of this survey reveal that the coexistence ratios of the two parasitoids on the three different positions on tobacco plants were higher than the individual existence ratios on them (Figure 1), which confirmed the measurement of spatial niche proportion similarity between the two parasitoids in the tobacco fields.

**Table 1:**
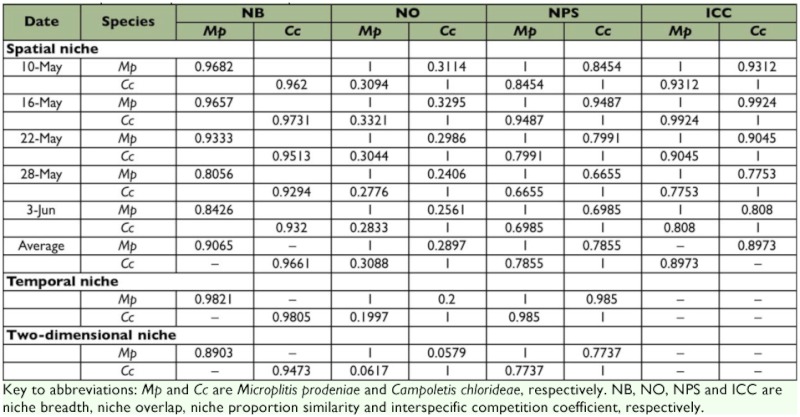
Temporal and spatial niches of *M. prodeniae* and *C. chlorideae* in the tobacco fields

The spatial niche breadth of *M. prodeniae* decreased with growing of tobacco, except in the case of the last observation (3 June). This was the same as for *C. chlorideae*, but exceptions were noticed on 3 June and 16 May. In addition, the spatial niche breadths of *M. prodeniae* were lower than those of *C. chlorideae,* but an exception was observed on 10 May. The results indicated that the active scopes of the two species in the early growth stages of tobacco were more extensive than those in the later growth stages of tobacco, and the active scope of *M. prodeniae* was smaller than that of *C. chlorideae*. The spatial niche overlap values of *M. prodeniae* were lower than those of *C. chlorideae*, suggesting that *M. prodeniae* was always a passive species when the spatial niche overlap occurred between the two species. In general, both the spatial niche proportion similarity and the interspecific competition coefficient between the two species were high during these experiments, revealing that the competition often happened between the two species when they occupied the same spatial resources in different stages. The spatial niche proportion similarity and interspecific competition coefficient between the two species were higher in the early growth stages than in the late growth stages of tobacco. The results show that severe competition between the two species happened in the early growth stages of tobacco (Table 1).

### Trophic niche of *M. prodeniae* and *C. chlorideae*

The trophic niche breadth of *M. prodeniae* was only 0.3333, but that of *C. chlorideae* was 0.8667, suggesting that the host range of *M. prodeniae* was narrower than that of *C. chlorideae* (Table 2). At the same time, the results of the survey also revealed that the parasitic rates of *M. prodeniae* on *S. litura* were 13.75 % – 48.75 %, and the parasitic rate of *M. prodeniae* on *H. assulta* and *H. armigera* were 0; however, the three lepidopterous host pests could be parasitized by *C. chlorideae* (Table 3), which is in accordance with the trophic niche breadths of the two species. The trophic niche overlap value of *M. prodeniae* was lower than that of *C. chlorideae*, and the results show that *M. prodeniae* existed in an inferior position when trophic niche overlap occurred between the two species. The trophic niche proportion similarity and the interspecific competition coefficient between the two species were 0.4054 and 0.6537, respectively. The results suggest that competition, to a certain extent, may happen between the two species when they compete for each other's food resources (Table 2).

The trophic niche breadth of *C. chlorideae* revealed a higher value than *M. prodeniae* in different stages, and the results indicate that the host range of *C. chlorideae* was wider than that of *M. prodeniae* in the tobacco fields. The trophic niche overlap values of *C. chlorideae* were superior to those of *M. prodeniae* in different stages, suggesting that *C. chlorideae* was dominant when the two species competed for food resources. The two species had greater trophic niche proportion similarity and the interspecific competition coefficients on 1 June and 7 June, respectively, indicating that severe competition happened between the two species over food resources during the late growth stages of tobacco compared with the early growth stages of tobacco in the tobacco fields (Table 2).

## Discussion

The parasitoid wasps are some of the most useful natural enemies, and many species have been used in biological control (van Lenteren et al. 1997). Both *M. prodeniae* and *C. chlorideae* are major parasitoids in the tobacco fields, and they were found to be significantly effective in reducing the natural population of *S. litura* (Dang and Hung 1999; Liu et al. 2004; Bajpai et al. 2005).

**Table 2:**
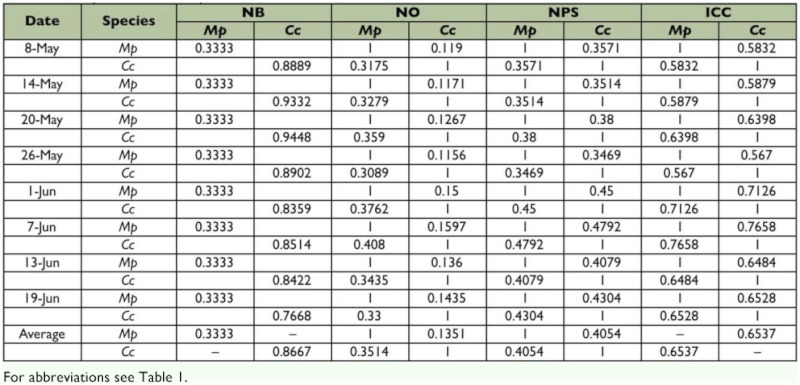
Trophic niches of *M. prodeniae* and *C. chlorideae* in the tobacco fields

**Table 3:**
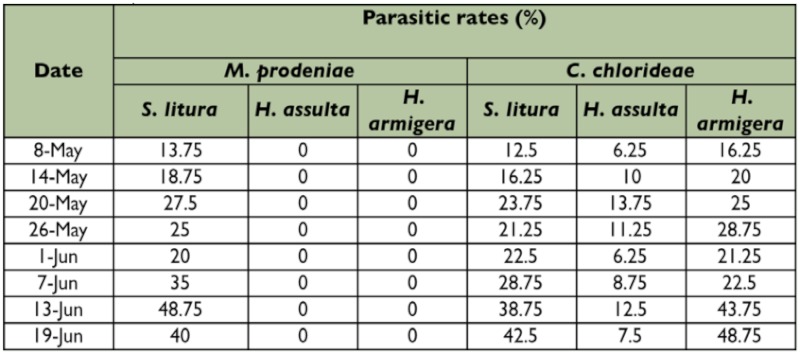
Parasitism of *M. prodeniae* and *C. chlorideae* on the three different host larvae in the tobacco fields

Those species that live in the same ecological spatial resource often conflict for occupying more food resources, especially when food resources are in short supply (Wissinger 1992; Denno et al. 1995; Stewart 1996; Reitz and Trumble 2002). In order to define the relationships between *M. prodeniae* and *C. chlorideae*, and understand whether competition between the two species occurs, niche and competition relationships of the two species were studied. The results showed that when the time and space in which the two species occurred was very similar, severe competition occurred between the two species for spatial resources in different stages, and that *M. prodeniae* was the passive species when the two competed for spatial resources. Zhou et al. (2006) considered that the spatial competition between two species was in proportion to the coexistence ratios of the two species on different positions of crop plants, but the intensity of competition may vary inversely with the individual existence ratios of the species. Because the coexistence ratios of the two species of parasitoids were dominant in tobacco plants, spatial competition between them would happen in tobacco fields.

In addition, the host range of *C. chlorideae* was wider than that of *M. prodeniae*, and competition would occur between the two species for occupying food resources. In particular, interspecific competition between the two species of parasitoids at the late stages was very severe. The populations of two parasitoids were enhanced with the increasing densities of their hosts (Zhou et al. 2007). The host species, *S. litura*, maintained a high population (Zhou et al. 2006), and they were concentrated within a certain of the tobacco plant when the yellow leaves were reaped from the bottom up during the late stages in the tobacco fields. As a result, the two parasitic species were also more concentrated during the late stages than those at the early stages of tobacco plant growth. This phenomenon where the population of the parasitoids is enhanced by the increasing densities of their hosts has been confirmed by previous studies (Cardinale et al. 2003; Cronin 2003; Garcia-Medel et al. 2007). Consequently, these results suggest that the intensity of competition between the two parasitoids will change when the densities and distributions of their hosts vary, and the increasing competition between the two parasitoids can be ascribed to the concentricity of their hosts in tobacco plants.

From these conclusions, it is suggested that the two species often conflict over spatial resources and food resources and that the competitive abilities of *C. chlorideae* to control spatial and food resources were better than those of *M. prodeniae.* Thus *C. chlorideae* may drive *M. prodeniae* away when it uses the same spatial or food resource. The competitive ability of *C. chlorideae* may be more greater when compared with *M. prodeniae* because the body size of a *C. chlorideae* adult (5.2 – 5.4 mm) is larger than that of an *M. prodeniae* adult (3.2–3.5 mm). Tian et al. (2008) reported that first instars of *Microplitis mediator* could physically attack the larvae of *C. chlorideae* because when host larvae were parasitized simultaneously by *M. mediator* and *C. chlorideae*, the majority of the cocoons produced were of *M. mediator.* However, whether the larvae of *M. prodeniae* could be physically attacked by the larvae of *C. chlorideae*, has yet to be determined.

According to the trophic niche breadths of the two species, the host range of *C. chlorideae* was wider than that of *M. prodeniae.* In general, *M. prodeniae* could only parasitize *S. litura* larvae, but *C. chlorideae* could parasitize the larvae of three major lepidopterous pests, *S. litura*, *H. assulta* and *H. armigera* in the tobacco fields (Kumar et al. 2000; Murugan et al. 2000; Liu et al. 2004; Pandey et al. 2004; Yang et al. 2005; Yan and Wang 2006; Zhang et al. 2006). Consequently, the adaptability of *C. chlorideae* to the environment in tobacco fields was better than that of *M. prodeniae*, and *C. chlorideae* retained a higher population compared with *M. prodeniae*. The results of these experiments suggest that the control of insect pests with *C. chlorideae* is feasible when *S. litura*, *H. assulta* and *H. armigera* occur together in the fields.

## References

[bibr01] Aquilino KM, Cardinale BJ, Ives AR (2005). Reciprocal effects of host plant and natural enemy diversity on herbivore suppression: an empirical study of a model tritrophic system.. *Oikos*.

[bibr02] Bajpai NK, Ballal CR, Rao NS, Singh SP, Bhaskaran TV (2005). Competitive interaction between two ichneumonid parasitoids of *Spodoptera litura*.. *Biocontrol Science and Technology*.

[bibr03] Begon M, Harper JL, Townsend CR (1996). *Ecology: Individuals, Populations and Communities.*.

[bibr04] Cardinale BJ, Harvey CT, Gross K, Ives AR (2003). Biodiversity and biocontrol: emergent impacts of a multienemy assemblage on pest suppression and crop yield in an agroecosystem.. *Ecology Letters*.

[bibr05] Cowll RK, Futuym DJ (1971). On the measurement of niche breadth and overlap.. *Ecology*.

[bibr06] Cronin JT (2003). Patch structure, oviposition behavior, and the distribution of parasitism risk.. *Ecological Monographs*.

[bibr07] Dang TD, Hung QC (1999). Composition of parasitic insects of soybean cutworm and eco-biological characteristics of *Microplitis prodeniae* Rao *et* Chandry (Hymenoptera: Braconidae) parasitic on *Spodoptera litura* F. (Lepidoptera: Noctuidae) in Hanoi and surrounding areas in Vietnam.. *Malaysian Applied Biology*.

[bibr08] Denno RF, McClure MS, Ott JR (1995). Interspecific interactions in phytophagous insects— competition reexamined and resurrected.. *Annual Review of Entomology*.

[bibr09] Duyck PF, David P, Quilici S (2004). A review of relationships between interspecific competition and invasions in fruit flies (Diptera: Tephritidae).. *Ecological Entomology*.

[bibr10] García-Medel D, Sivinski J, Díaz-Fleischer F, Ramirez-Romero R, Aluja M (2007). Foraging behavior by six fruit fly parasitoids (Hymenoptera: Braconidae) released as single- or multiple-species cohorts in field cages: Influence of fruit location and host density.. *Biological Control*.

[bibr11] Geoff G, Steve W (2000). *Biological Control: Measures of Success.*.

[bibr12] Kruess A, Tscharntke T (1994). Habitat fragmentation, species loss, and biological control.. *Science*.

[bibr13] Kumar N, Kumar A, Tripathi CPM (2000). Sex ratio of *Campoletis chlorideae* Uchida in response to *helicoverpa armigera* (Hübner) density.. *Insect Science and Its Application*.

[bibr14] Levins R (1968). *Evolution in Changing Environment.*.

[bibr15] Liu WX, Wan FH, Yuan ST (2004). Massrearing and bionomics of *Campoletis chlorideae*.. *Chinese Journal of Biological Control*.

[bibr16] Mackauer M, Mackauer M, Ehler LE, Roland J (1990). Host discrimination and larval competition in solitary endoparasitoids.. *Critical Issues in Biological Control*.

[bibr17] May RM (1975). Some notes on estimating the competition matrix.. *Ecology*.

[bibr18] Murugan K, Kumar NS, Jeyabalan D, Nathan SS, Slvaramakrishnan S, Swamiappan M (2000). Influence of *Helicoverpa armigera* (Hübner) diet on its parasitoid *Campoletis chlorideae* Uchida.. *Insect Science and Its Application*.

[bibr19] Pandey P, Kumar N, Tripathi CPM (2004). Impact of males on the progeny sex ratio of *Campoletis chlorideae* (Hymenoptera: Ichneumonidae), a parasitoid of *Helicoverpa armigera* (Hübner) (Lepidoptera: Noctuidae).. *Journal of Applied Entomology*.

[bibr20] Pimentel D (1961). Species diversity and insect population outbreaks.. *Annals of the Entomological Society of America*.

[bibr21] Qian LW, Wu CZ, Hong W (2006). Ecological niche in dominant species in *Tsuga longibracteata* forest gaps with different development stages.. *Journal of Tropical and Subtropical Botany*.

[bibr22] Reitz SR, Trumble JT (2002). Competitive displacement among insects and arachnids.. *Annual Review of Entomology*.

[bibr23] Shorrocks B (1991). Competition on a divided and ephemeral resource: a cage experiment.. *Biological Journal of The Linnean Society*.

[bibr24] Stewart AJA (1996). Interspecific competition reinstated as an important force structuring insect herbivore communities.. *Trends in Ecology and Evolution*.

[bibr25] Tian SP, Zhang JH, Yan YH, Wang CZ (2008). Interspecific competition between the ichneumonid *Campoletis chlorideae* and the braconid *Microplitis mediator* in their host *Helicoverpa armigera*.. *Entomologia Experimentalis et Applicata*.

[bibr26] van Lenteren JC (1997). Benefits and risks of introducing exotic macro-biological control agents into Europe.. Bulletin OEPP/EPPO.

[bibr27] Vinson SB, Iwantsch GF (1980). Host suitability for insect parasitoids.. *Annual Review of Entomology*.

[bibr28] Wilb A, Thomas MB (2002). Natural enemy diversity and pest control: patterns of pest emergence with agricultural intensification.. *Ecology Letters*.

[bibr29] Wissinger SA (1992). Niche overlap and the potential for competition and intraguild predation between size-structured populations.. *Ecology*.

[bibr30] Yan ZG, Wang CZ (2006). Similar attractiveness of maize volatiles induced by *Helicoverpa armigera* and *Pseudaletia separata* to the generalist parasitoid *Campoletis chlorideae*.. *Entomologia Experimentalis et Applicata*.

[bibr31] Yang YZ, Yu YS, Ren L, Shao YD, Qian K, Zalucki MP (2005). Possible incompatibility between transgenic cottons and parasitoids.. *Australian Journal of Entomology*.

[bibr32] Zhang SY, Xie BY, Cui J, Li DM (2006). Biology of *Campoletis chlorideae* (Uchida) (Hymenoptera: Ichneumonidae) developing in Bt-treated, Bt-resistant *Helicoverpa armigera* (Hübner) (Lepidoptera: Noctuidae) larvae.. *Journal of Applied Entomology*.

[bibr33] Zhou F, Fang HL (2000). On the interspecific niche relationship between two species of wren warbler.. *Zoological Research*.

[bibr34] Zhou ZS, Chen ZP, Xu ZF (2006). Niches of *Spodoptera litura* (Fabricius) and *Helicoverpa assulta* (Guenée) in tobacco plants.. *Acta Ecologica Sinica*.

[bibr35] Zhou ZS, Chen ZP, Deng HB, Chen YM, Xu ZF (2007). Life table of natural population of *Spodoptera litura* (Fabricius) on tobacco and taro.. *Acta Ecologica Sinica*.

